# α-Carboline derivative TJY-16 inhibits tumor growth by inducing G2/M cell cycle arrest in glioma cells

**DOI:** 10.1186/s12929-016-0222-y

**Published:** 2016-01-19

**Authors:** Hsiao-Chieh Huang, Wei-Ting Liu, Kuo-Su Hua, Hui-Chi Hung, Jui-Ying Tsai, Sheng-Chu Kuo, Li-Jiau Huang, Po-Wu Gean

**Affiliations:** Department of Pharmacology, College of Medicine, National Cheng Kung University, Tainan, Taiwan; Graduate Institute of Pharmaceutical Chemistry, China Medical University, Taichung, Taiwan

**Keywords:** Glioblastoma multiforme, Carboline derivatives, Cell cycle arrest, Apoptosis, G_2_/M arrest

## Abstract

**Background:**

Glioblastoma multiforme (GBM) is the most lethal primary brain tumors which remains difficult to cure despite advances in surgery, radiotherapy and chemotherapy. Therefore, the development of new drug is urgently needed. α-carboline derivatives were usually isolated from marine animals such as Britannia marine tunicate *Dendrodoa grossularia* and Indonesian ascidian *Polycarpa aurata*. In this study, we have synthesized several α-carboline compounds and examined their anti-glioma activities.

**Results:**

We report that among α-carboline derivatives TJY-16 (6-acetyl-9-(3,4,5-trimethoxybenzyl)-9H-pyrido[2,3-b] indole) is the most potent α-carboline analog to induce glioma cell death with IC_50_ value of around 50 nM. TJY-16 decreased cell viability of glioma cells in a concentration- and time-dependent manner. Trypan blue exclusion assay showed that the reduction of cell viability was due to both cell growth inhibition and cell death. Flow cytometric analysis showed that TJY-16 induced G2/M cell cycle arrest followed by induction of sub-G1 phase. Hoechst staining detected the apoptotic features such as nuclear shrinkage and DNA condensation. Western blot analysis showed the increased level of cleaved caspase-3. The activation of caspase-8 and depolarization of mitochondrial membrane potential (ΔΨm) indicated that both extrinsic and intrinsic apoptotic pathways were involved in TJY-16-induced apoptosis. TJY-16 effectively inhibited tumor growth and induced caspase-3 activation in the xenograft tumor model of U87 glioma cells.

**Conclusions:**

Our results suggest that TJY-16 may kill glioma cells by inducing G2/M cell cycle arrest followed by apoptosis. Thus, TJY-16 is a promising agent for the treatment of malignant gliomas.

## Background

Glioblastoma multiforme (GBM), an astrocytic origin of glioma, is among the most common and malignant subtype [1]. Despite the relative low incident rate, the highly invasive nature of GBM still leads to a disproportionately high mortality. After receiving optimal treatment, the median survival rate of patients bearing GBM is still less than 15 months [2, 3] with a 5-year survival rate of less than 5 % [4]. Temozolomide, a Blood Brain Barrier-permeable DNA alkylating agent [5], is the first line chemotherapy for treating GBM nowadays. Unfortunately, recurrence of GBM after standard therapies is almost inevitable [6, 7] highlighting an imperative need to develop novel strategies for the treatment of GBM.

Carboline is a nitrogen-containing heterocyclic compound that consists of two isoforms:α-carboline and β-carboline. β-carboline alkaloids have been found to exhibit several pharmacological effects like anti-cancer [8–10], anti-malaria [11], anti-dopaminergic activity [12]. In contrast, the studies of α-carboline derivatives were limited [13, 14]. α-carboline derivatives were usually isolated from marine animals such as Britannia marine tunicate *Dendrodoa grossularia* ^14^ and Indonesian ascidian *Polycarpa aurata* [15]. We have synthesized a series of α-carboline compounds in which TJY-16 (previously termed HAC-Y6) has been shown to display potent anti-cancer activity against hepatocellular carcinoma cells (HCC) in vitro through disrupting microtubule assembly, causing cell cycle arrest and apoptosis [16]. In this study, we examined their anti-glioma activities.

Cell cycle is a complex process controlled by numerous regulatory proteins that ensure the accuracy of DNA replication and division [17, 18]. Dysregulation of cell cycle and cellular proliferation causes unrestrained cell growth and cancer development [19]. The G_2_ checkpoint allows the cell to repair DNA damage before entering mitosis. Defects in the G_2_/M arrest checkpoint may allow a damaged cell to enter mitosis and undergo apoptosis, and efforts to enhance this effect may increase the cytotoxicity of chemotherapy. In the present study, we investigated the effect of synthetic α-carboline derivatives on malignant glioma cells. We found that TJY-16-induced cell death was associated with G_2_/M cell cycle arrest followed by induction of sub-G1 phase. Hoechst staining detected the nuclear shrinkage and DNA condensation that were typical characteristics of apoptosis. TJY-16 effectively inhibited tumor growth and induced apoptosis in the xenograft animal model of U87 human glioma cells. Thus, TJY-16 seems to be a promising agent for the treatment of malignant gliomas.

## Methods

### Cell culture and reagents

The human glioma cell lines U87, U251, T98G provided by Dr. Michael Hsiao (Genomics Research Center, Academia Sinica, Taiwan) were cultured in Dulbecco’s Modified Eagle medium (DMEM, Caisson) supplemented with 10 % fetal bovine serum (FBS, Sigma-Aldrich), 2 mM L-glutamine (Caisson), 100 U/ml penicillin, and 0.1 mg/ml streptomycin (Caisson). The rat glioma C6 cell line provided by Dr. Shun-Fen Tzeng (National Cheng Kung University, Taiwan) was cultured in DMEM/F12 (Caisson) supplemented with 10 % fetal bovine serum, 2 mM L-glutamine, 100 U/ml penicillin, and 0.1 mg/ml streptomycin. The human normal glia cell line SVGP12, kindly provided by Dr. Michael Hsiao was cultured in Minimum Essential Medium (MEM, Invitrogen) supplemented with 10 % fetal bovine serum, 2 mM L-glutamine, 100 U/ml penicillin, and 0.1 mg/ml streptomycin. All cells were maintained in a humidified atmosphere containing 5 % CO_2_ at 37 °C. TJY-13, TJY-14, TJY-16, TJY-18, TJY-22, TJY-24 provided by Dr. Li-Jiau Huang (China Medical University, Taiwan) were dissolved in dimethylsulfoxide (DMSO).

### Animal study

All mice in this study were BALB/cAnN.Cg-*Foxn1*^*nu*^/CrlNarl mice purchased from the National Laboratory Animal Center (NLAC). Mice were housed in individual plastic cages with controlled temperature (22 ± 2 °C) and humidity (55 ± 5 %) and were kept on a 12 h/12 h light/dark cycle, and were given free access to water and food. Care and use of laboratory animals were in accordance with National Institutes of Health (NIH) guidelines. All procedures were approved of the Institutional Animal Care and Use Committee of the College of Medicine, NCKU with project approval number (#103041).

For tumorigenesis, U87 glioma cells (1 × 10^6^ cells in l00 μL PBS) were inoculated subcutaneously into the right flank of 8 to 10-week-old male nude mice (nu/nu BALB-c) which were purchased from National Laboratory Animal Center. Tumor volume was measured every three days and body weights were measured once per week. Tumor volume was calculated by volume(mm^3^) = (length × width^2^)/2 [20]. When the tumors reached a mean volume of 40 to 80 mm^3^, TJY-16 (24 mg/kg in saline) was injected intraperitoneally once per day for 10 days and TMZ (80 mg/kg in saline) was administered orally for 5 days.

### WST-1 assay

5x10^3^ glial cells or 2x10^3^ glioma cells per well were seeded in 96-well plates and allowed to attach overnight at 37 °C. Culture medium containing vehicle or drugs was added to the medium in each well, and cells were incubated at 37 °C for indicated time points. Cell proliferation was determined by WST-1 which is a colorimetric assay of cell viability that is based on the enzymatic cleavage of the tetrazolium salt WST-1 by mitochondrial dehydrogenases. The water-soluble formazan dye can be detected by absorbance at 420–480 nm. At indicated time points, medium were removed, and then fresh culture medium (100 μL/well) with WST-1 solution (10 μL/well) were added. After the plate was incubated for 0.5–4 h at 37 °C, the absorbance of soluble formazan was measured at 440 nm with a microplate reader (Molecular device). Cell viability was presented as the percentage of survival relative to vehicle-treated control.

### Trypan-blue exclusion assay

Cell viability was also determined by trypan-blue exclusion assay. Trypan-blue (Sigma) exclusion assay is a simple and rapid method measuring cell viability that determines the number of viable cells and dead cells. It is based on the principle that live cells with an intact membrane are able to exclude the dye whereas dead cells without an intact membrane take up the dye. 2x10^4^ cells per well were seeded in 6-well plates and allowed to attach overnight at 37 °C. Culture medium containing vehicle or drugs was added to each well, and cells were incubated at 37 °C. At indicated time points (0, 24, 48, 72, 96 h), cells were suspended with 0.05 % trypsin-EDTA, and stained with trypan-blue dye (0.4 %). The unstained (viable) and stained (dead) cells were separately counted in the hemocytometer. The cell growth curve was determined by live cells relative to the total number of cells, and cell death (%) was evaluated by the percentage of death relative to the total cells.

### Cell cycle analysis

1x10^5^ glioma cells per well were seeded in 6-well plates and allowed to attach overnight at 37 °C. Culture medium containing vehicle or drugs was added to the medium in each well, and cells were incubated at 37 °C for indicated time points. At indicated time points, cells were trypsinized and suspended with ice cold PBS and fixed in 70 % ethanol at −20 °C overnight. After fixation, the cells were washed twice with ice cold PBS and were centrifuged at 1,000 rpm for 5 min at 4 °C. Finally, the cells were resuspended in 1 ml PI/Triton-X 100 solution (0.1 % Triton-X 100, 0.2 mg/ml RNase A and 20 μg/ml propidium iodide in PBS) for 30 min in the dark. Cell cycle distribution was analyzed from 10,000 cells in a FacScan flow cytometer (BD Biosciences). The percentage of cells in different phases was analyzed by WinMDI software.

### Detection of mitochondrial membrane potential by JC-1 staining

1x10^5^ glioma cells per well were seeded in 6-well plates and allowed to attach overnight at 37 °C. Culture medium containing drugs was added to the medium in each well, and cells were incubated at 37 °C for indicated time points. At indicated time points, cells were collected by centrifugation for 5 min at 400 × g at 4 °C and resuspended in JC-1 (5,5’,6,6’-tetrachloro- 1,1’,3,3’-tetraethylbenzimidazolcarbocyanine iodide, BD Biosciences) working solution. The cells were incubated for 10 ~ 15 min at 37 °C in CO_2_ incubator, washed twice with 1x assay buffer, resuspended in 500 μl 1x assay buffer, and analyzed immediately by FacScan flow cytometer (BD Biosciences). JC-1 fluorochrome is rapidly taken up by healthy mitochondria (polarized), leading to the formation of JC-1 aggregates which emit red fluorescence at 590 nm. However, JC-1 does not accumulate in unhealthy mitochondria (depolarized) and remains in the cytoplasm as monomers which emit green fluorescence at 527 nm. The changes in mitochondrial membrane potential were measured by the ratio of the red to green JC-1 fluorescence.

### Hoechst Staining

5x10^4^ glioma cells per well were seeded in 12-well plates and allowed to attach overnight at 37 °C. Culture medium containing drugs was added to the medium in each well, and cells were incubated at 37 °C for 24 h. At 24 h, the supernatant was discarded and the cells were washed twice with ice cold PBS. Then cells were fixed with 4 % paraformaldehyde in PBS for 10 min at room temperature. After washing twice with PBS, cells were incubated with Hoechst 33342 (0.1 μg/ml, Sigma-Aldrich) for 10 min at room temperature in the dark. Fluorescence images were detected by Leica DM IL microscope with NIKON P6000 camera.

### Western blotting assay

6x10^5^ cells were seeded in 10 cm plates and allowed to attach overnight at 37 °C. Glioma cells were treated with medium containing TJY-16 50 nM or vehicle. At indicated time, cell pellets were collected and centrifuged at 4,000 rpm and stored at −20 °C. Drugs- or vehicle-treated cell pellets were lysed in a lysis buffer containing 50 mM Tris–HCl, pH 7.4, 150 mM NaCl, 1 % Nonidet P-40, 0.25 % sodium deoxycholate, 0.1 % SDS, protease inhibitor (Roche) and phosphatase inhibitor (Roche). After lysates centrifuged at 13000 rpm for 30 min at 4 °C, supernatants were collected, loaded on the wells of SDS-polyacrylamide gel. Then, transferred separated proteins to a PVDF membrane (Immunobilon transfer membranes, Millipore) by semi-dry transfer system (BIO-RAD). The membrane was then immersed in 5 % nonfat dry milk for 60 min at room temperature followed by reacting with primary antibodies:rabbit monoclonal caspase-3, rabbit monoclonal caspase-8, rabbit monoclonal BID (Cell Signaling Technology), mouse monoclonal α-tubulin (Sigma-Aldrich), mouse monoclonal β-actin (Millipore) at 4 °C overnight. Then the membrane was incubated with HRP-conjugated secondary antibodies (Jackson ImmunoResearch Lab., USA) for 1 h. Chemiluminescent detection was performed using the western blot chemiluminescence reagent system (Perkin-Elmer, NEL103001EA). X-ray films were exposed at different time points to ensure the optimum density, but not saturated.

### Statistical analysis

Experiments were performed at least in triplicate. Data were presented as mean ± standard error of the mean (SEM). Independent experiments were analyzed by unpaired *t* test. P < 0.05 was considered statistically significant.

## Results

### α-carboline derivatives inhibit glioma cell viability

**Fig. 1:**
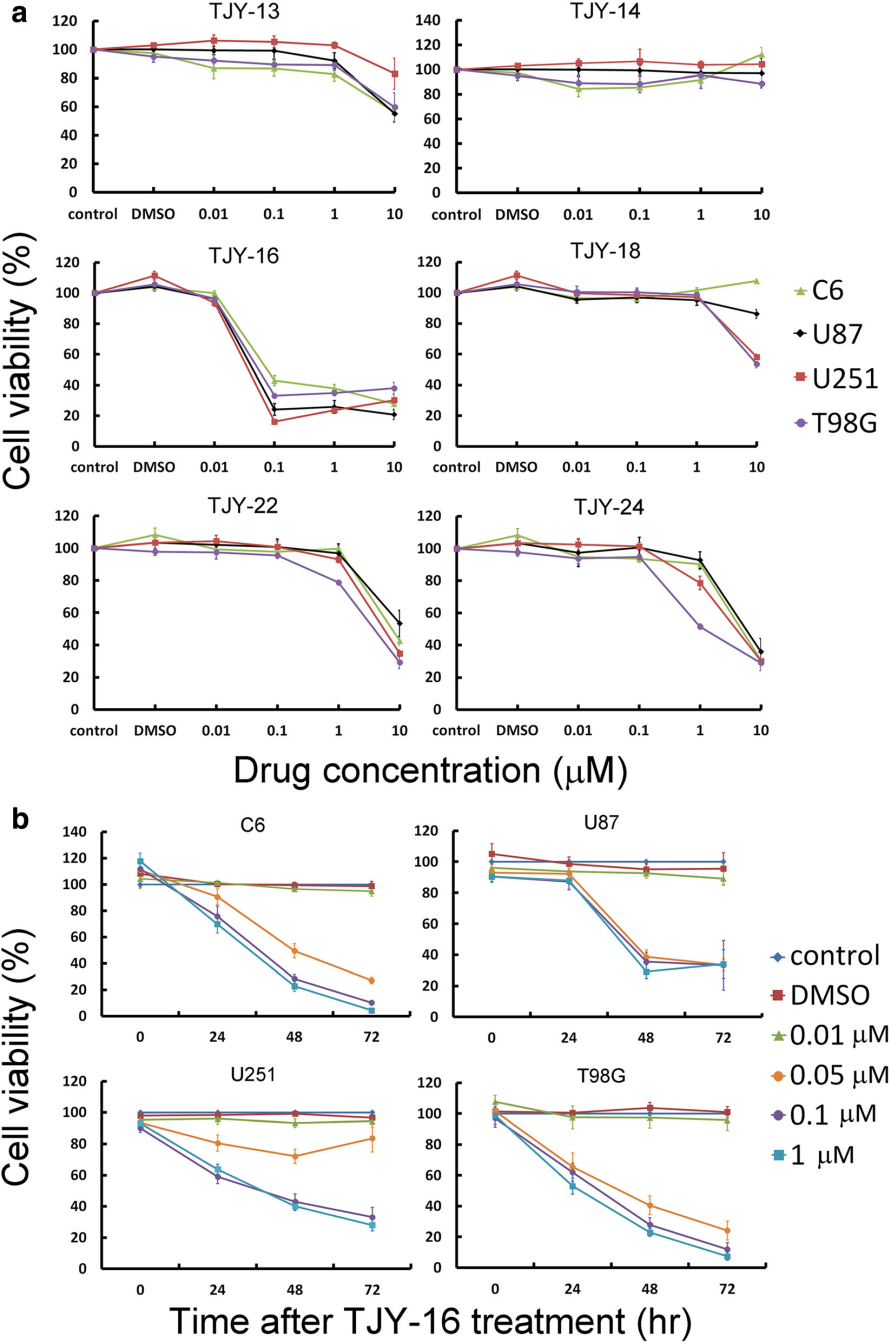
The effects of α-carboline derivatives on glioma cell lines. **a** Concentration-dependent effects of TJY-13, TJY-14, TJY-16, TJY-18, TJY-22 and TJY-24 in C6, U87, T98G, and U251 glioma cell lines. Cells were treated with various concentrations of drugs for 48 h and cell viability was determined by WST-1 assay. **b** Concentration- and time-dependent reduction of cell viability in C6, U87, T98G, and U251 glioma cells by TJY-16

To investigate the effects of α-carboline analogs on cell proliferation, C6 rat glioma cells and U87, T98G and U251 human glioma cells were treated with various concentrations of TJY-13, TJY-14, TJY-16, TJY-18, TJY-22 and TJY-24 for 48 h and cell viability was assessed by WST-1 assay. As shown in Fig. 1a, the cell viability was effectively inhibited by α-carboline analogs. Table 1 showed the structure of TJY-16 and the IC_50_ values of α-carboline analogs and IC_50_ value of TJY-16 was comparably lower than other α-carboline analogs. Since TJY-16 was the most potent compound, we further investigated its concentration- and time-dependent effects on C6, U87, T98G and U251 glioma cells (Fig. 1b).

**Table 1:**
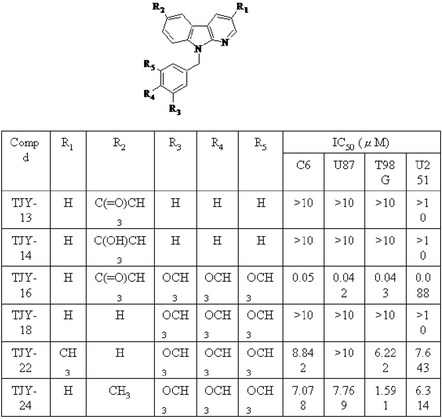
Structures and IC_50_ values of α-carboline derivatives against glioma cells

**Fig. 2:**
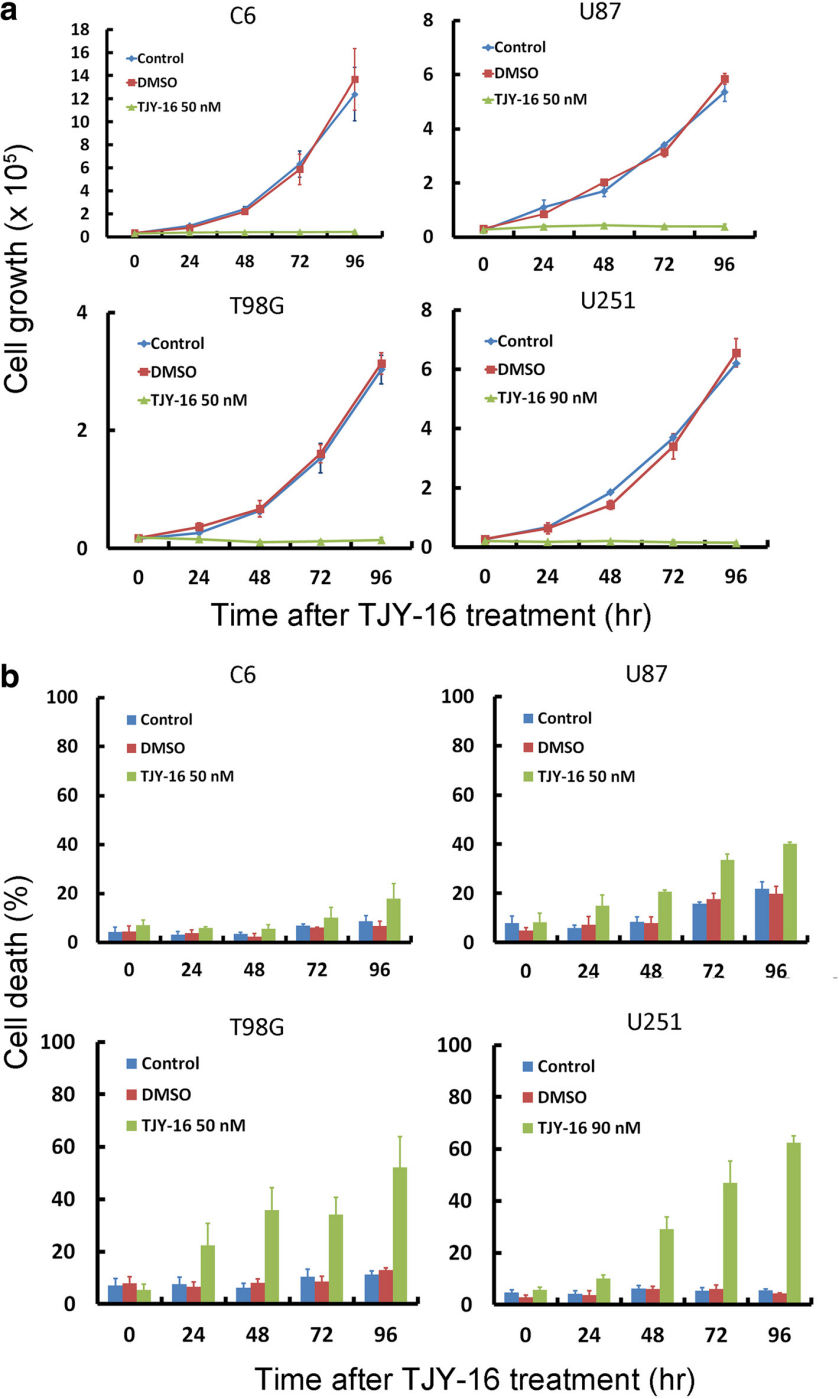
Effects of TJY-16 on glioma cell growth and cell death. **a** C6, U87, T98G and U251 glioma cells were treated with 50 nM TJY-16 for the indicated times and cell growth curve was determined by *trypan blue* exclusion assay. **b** C6, U87, T98G and U251 glioma cells were treated with 50 nM TJY-16 for the indicated times and cell death was determined by *trypan blue* exclusion assay. (mean ± SEM, *n* = 3). *P < 0.05, **P < 0.01, ***P < 0.001 vs. control

Trypan blue exclusion assay is frequently used to determine the number of viable and dead cells presented in a cell suspension [21]. As shown in Fig. 2a, TJY-16 treatment rmarkedly suppressed cell growth curve compared with control (without any treatment) or vehicle treatment in C6, U87, T98G, U251 glioma cell lines. In parallel, the percentage of cell death increased significantly (Fig. 2b).

### TJY-16 causes cell cycle arrest at G2/M phase in glioma cells

**Fig. 3:**
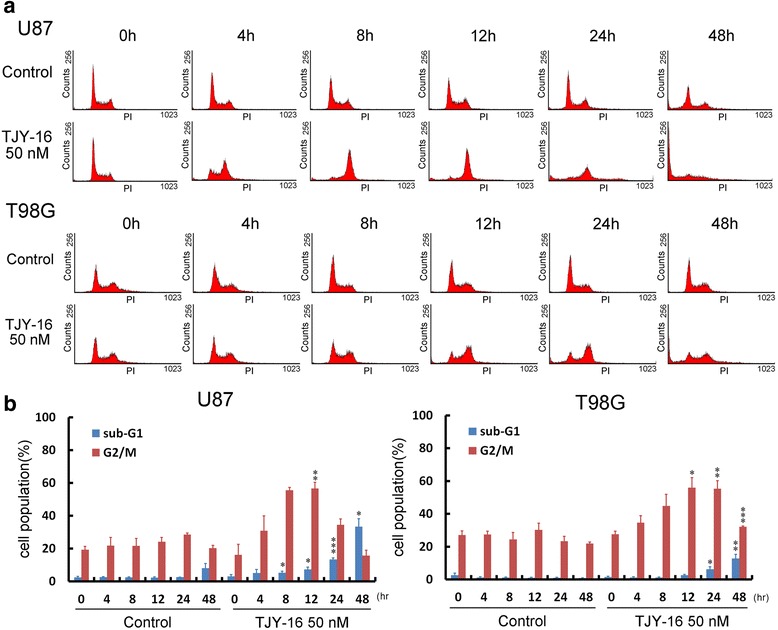
TJY-16 caused glioma cells G2/M arrest and cell death. **a** U87 and T98G glioma cells were treated with TJY-16 (50 nM) for the indicated times and cell cycle distributions were monitored by flow cytometry with propidium iodide staining. **b** Quantification of sub-G1 and G2/M phase were analyzed by WinMDI software (mean ± SEM, *n* = 3). *P < 0.05, **P < 0.01, ***P < 0.001 vs. control

Several studies have demonstrated that there are various molecular linkages between cell death and cell cycle arrest [19]. We examined the effect of TJY-16 on cell cycle distribution. U87, T98G glioma cells, treated with 50 nM TJY-16 for the indicated times, were stained with propidium iodide (PI) and cell cycle distribution was monitored by flow cytometry. FACS analysis revealed that 12 h of TJY-16 treatment significantly increased the percentage of cells in the G2/M phase (Fig. 3a). In addition, 24 to 48 h after TJY-16 treatment, the percentage of cells in the sub-G1 phase was significantly increased (Fig. 3a). The quantifications of sub-G1 and G2/M phase in U87 and T98G cells were shown in Fig. 3b.

### Both extrinsic and intrinsic apoptotic pathways are involved in TJY-16-induced cell death

**Fig. 4:**
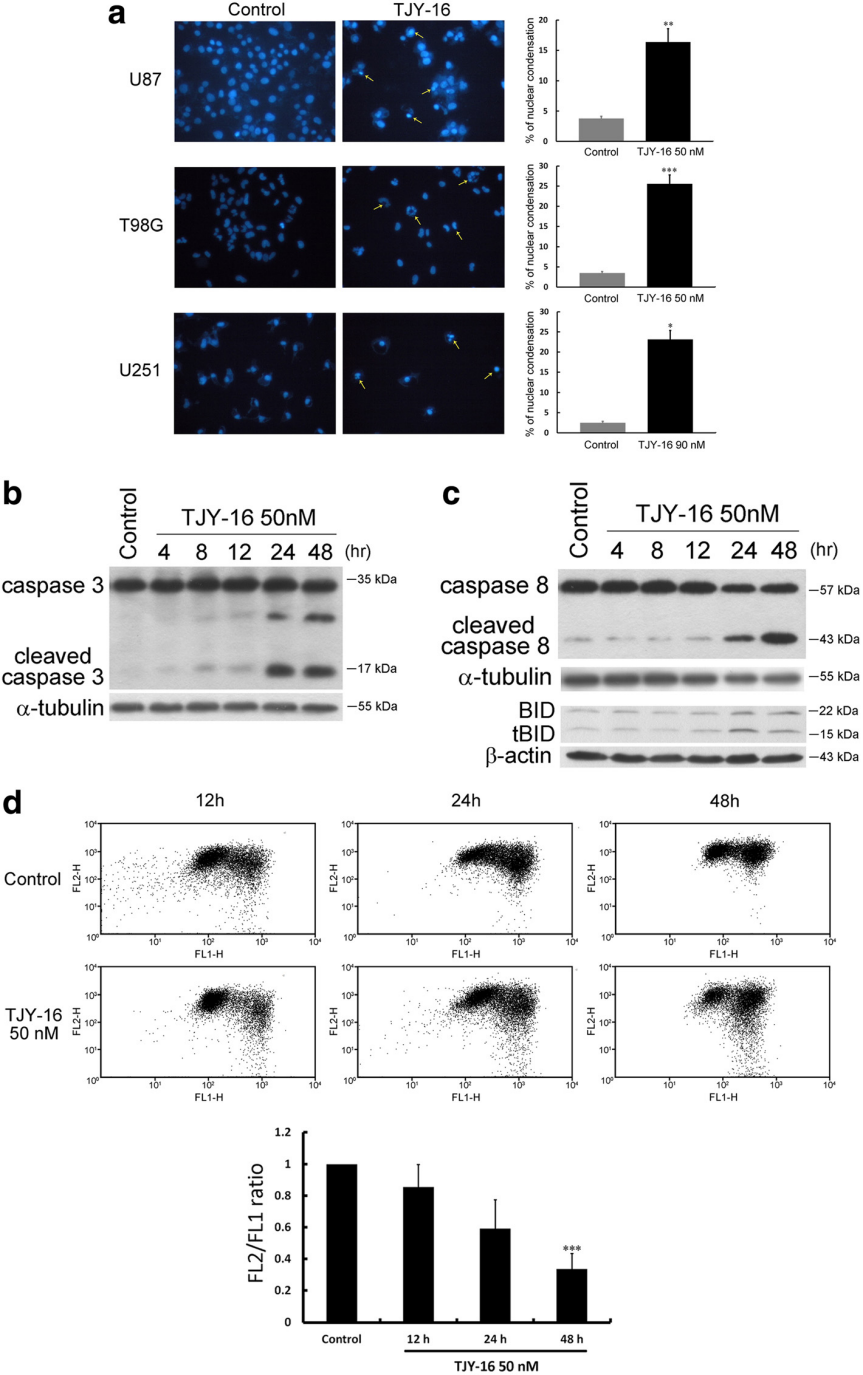
TJY-16-induced cell death involves both extrinsic and intrinsic apoptotic pathways. **a** U87, T98G and U251 glioma cells were treated with 50 nM TJY-16 for 24 h and morphology was analyzed with Hoechst 33342 staining. Apoptotic cells increased after the treatment with TJY-16. **b** Western blotting analysis of caspase-3 activation in U87 glioma cells treated with TJY-16 (50 nM) for the indicated times. **c** Western blotting analysis of caspase-8 and BID activation in U87 glioma cells treated with TJY-16 (50 nM) for the indicated times. **d** U87 glioma cells were treated with 50 nM TJY-16 for indicated times, mitochondrial membrane potential (ΔΨm) were measured with JC-1 dye and flow cytometry. ΔΨm changes were analyzed by the ratio of the *red* to *green* JC-1 fluorescence (bottom, mean ± SEM, *n* = 3). *P < 0.05, **P < 0.01, ***P < 0.001 vs. control

We used Hoechst staining to detect the characteristics of apoptosis. Microscopy images of TJY-16-treated cells showed apoptotic features such as nuclear shrinkage and DNA condensation (Fig. 4a). Moreover, treatment of TJY-16 for 24 h induced caspase-3 activation (Fig. 4b). Depending on the origin of stress, apoptotic response is through two major pathways: the extrinsic (death receptor) and the intrinsic (mitochondrial) pathways [22]. Once the extrinsic pathway is activated, procaspase-8 is cleaved which then triggers caspase-3 activation. Thus, we measured the protein level of caspase-8. Figure 4c revealed that cleaved caspase-8 was increased at 24 h after treatment with TJY-16 in U87 glioma cell. BID is a pro-apoptotic protein of the Bcl-2 family which is downstream of caspase-8 [23]. We found that truncated BID (tBID) also was increased in TJY-16-treated U87 glioma cell (Fig. 4c). In the intrinsic pathway, loss of mitochondrial membrane potential (ΔΨm) plays an important role which leads to release of cytochrome c and other pro-apoptotic factors. Therefore, we used JC-1 staining to detect mitochondrial membrane potential (ΔΨm) following TJY-16 treatment. As shown in Fig. 4d, TJY-16-treated cells had lower red/green ratio compared to control, indicating mitochondrial membrane potential (ΔΨm) was depolarized after treatment with TJY-16. As reduction of red/green ratio was considered to be an indicator of loss of mitochondrial membrane potential (ΔΨm), the quantification of ΔΨm was expressed as the ratio of red/green JC-1 fluorescence (Fig. 4d bottom). Taken together, these data implied that TJY-16-induced glioma cell death involved both extrinsic and intrinsic apoptotic pathways.

### TJY-16 inhibits growth of subcutaneous tumors by inducing apoptosis

**Fig. 5:**
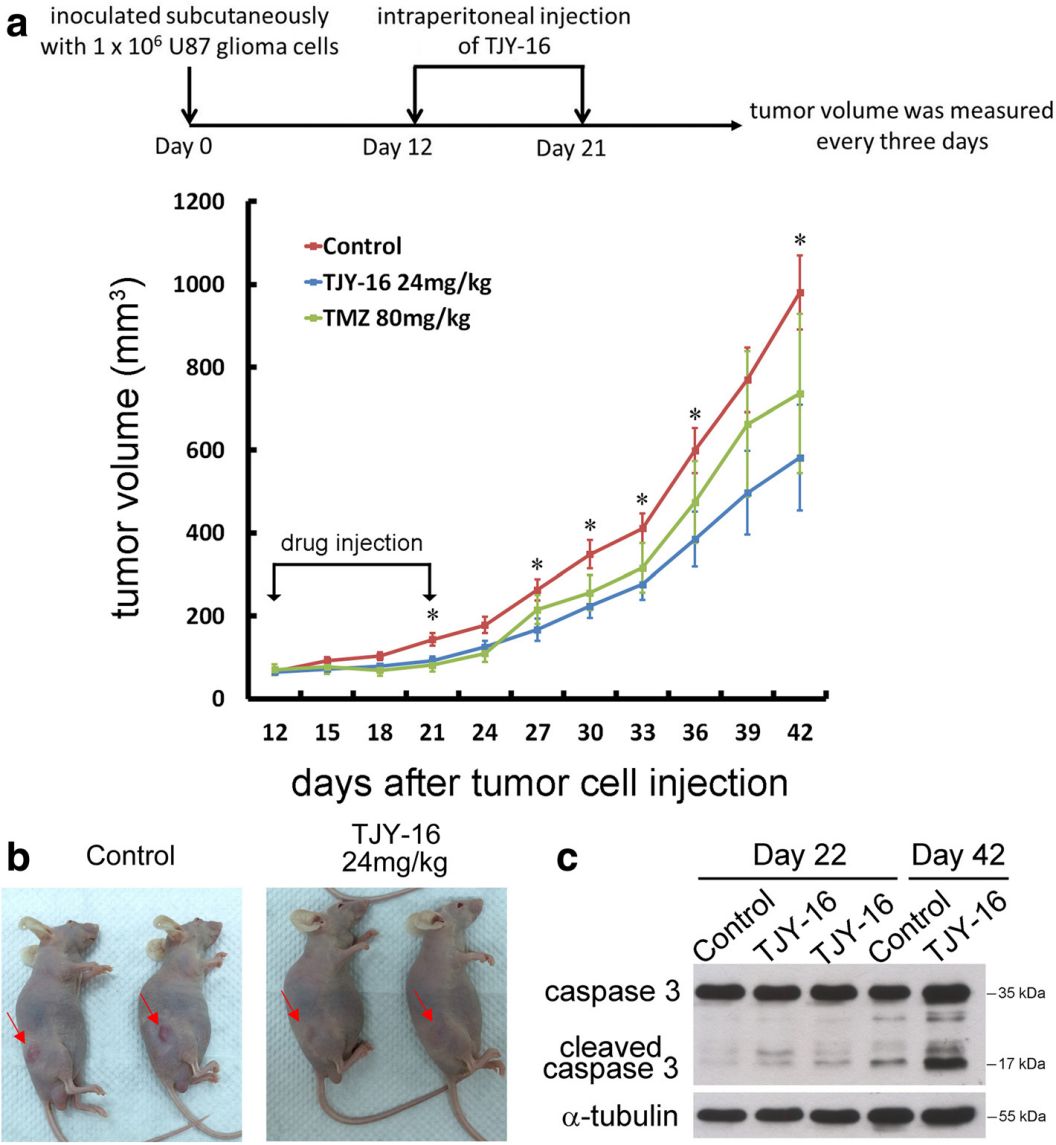
TJY-16 inhibits the growth of glioma cells in vivo by inducing apoptosis. **a** 1 × 10^6^ U87 glioma cells inoculated subcutaneously into the right flank of nude mice (*n* = 6 for each group). When the tumors reached 40 to 80 mm^3^ in volume, TJY-16 (24 mg/kg) were administered intraperitoneally every 24 h for 10 days and tumor volume was measured for another 20 days after the cessation of treatment. Temozolomide (TMZ, positive control) was given orally once per day for 5 days. *P < 0.05 vs. control. **b** Mice with xenograft U87 tumors were photographed after treatment with TJY-16 or control. **c** Western blots showed induction of caspase-3 activation at day 22 and day 42

**Fig. 6:**
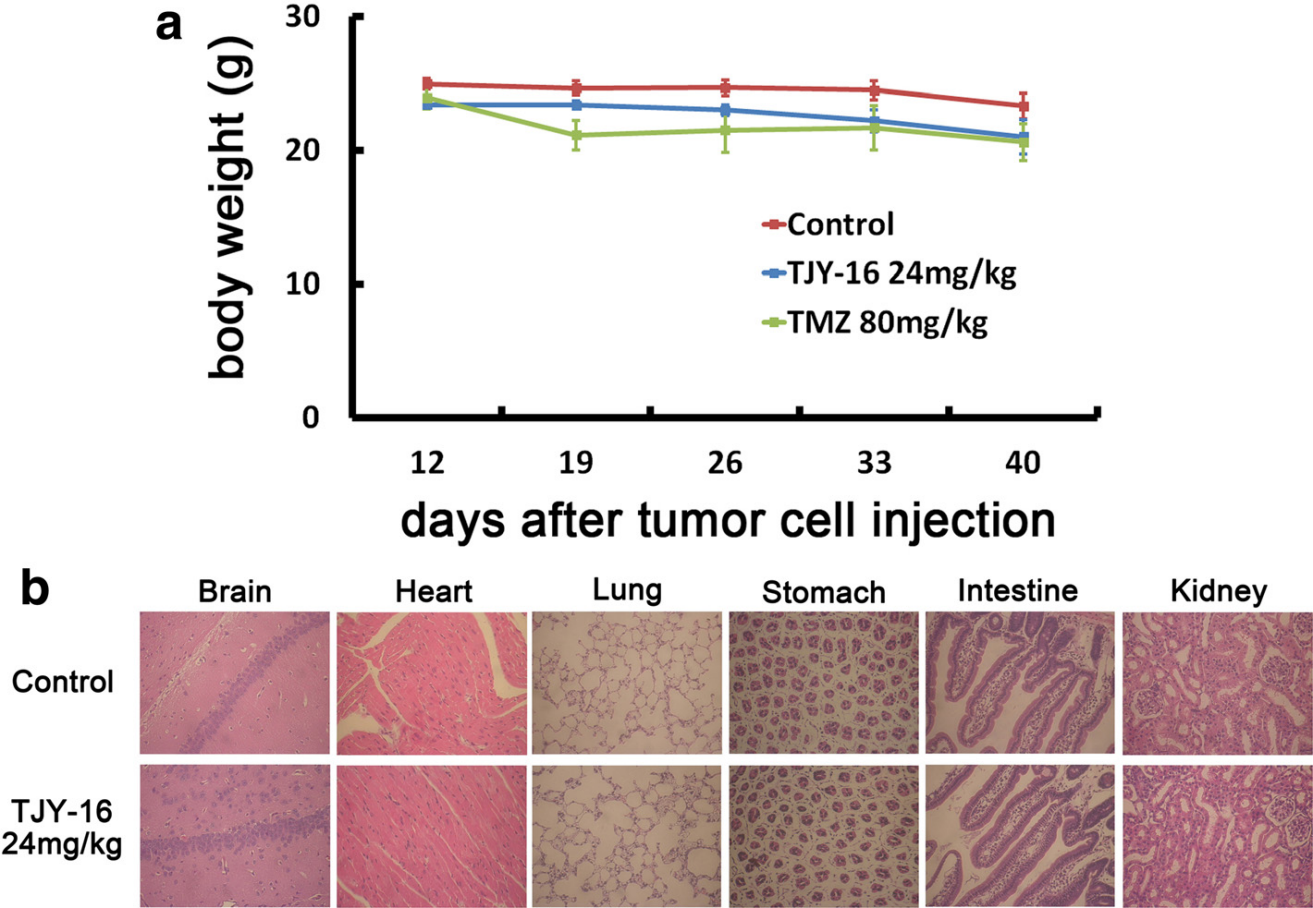
TJY-16 does not significantly influence body weight and cause organ toxicity. **a** The body weights of mice were measured once per week until the end of experiment. There were no significant differences between control and TJY-16-treated mice. **b** Hematoxylin and eosin (H&E) staining of tissue sections of brain (hippocampus), heart, lung, stomach, intestine and kidney from healthy (no inoculation nude mice) and TJY-16-treated tumor inoculated mice

We determined whether TJY-16 inhibited growth and induced apoptosis in U87 glioma cells in vivo. Nude mice were inoculated subcutaneously with 1 × 10^6^ U87 glioma cells. When tumor volume reached 40 to 80 mm^3^, TJY-16 (24 mg/kg) was injected intraperitoneally once per day for 10 days whereas TMZ (80 mg/kg) was orally administered once per day for 5 days. Tumor growth was observed for 20 days after the cessation of treatment. Twenty days after the cessation of drug injection, tumor growth was significantly inhibited by TJY-16 (Fig. 5a, 5b). To examine whether TJY-16 induced apoptosis in vivo, we examined the expression of cleaved caspase-3. As shown in Fig. 5c, expression of cleaved caspase-3 increased significantly in tumors treated with TJY-16. These results suggested that TJY-16-mediated tumor growth inhibition in vivo was associated with apoptotic cell death. TJY-16 did not affect the body weight of the mice (Fig. 6a). Furthermore, histological phenotypes in brain, heart, lung, stomach, intestine and kidney were not changed in TJY-16-treated mice compared to healthy mice (Fig. 6b).

## Discussion

In the present study, we aimed to evaluate the anti-glioma activity of novel α-carboline derivatives which were synthesized from the laboratory [24]. Using WST-1 assay, we found TJY-16 was the most potent α-carboline derivatives in inhibiting glioma cell lines. TJY-16 reduced cell viability of C6, U87, T98G and U251 glioma cells in a time- and concentration-dependent manner with IC_50_ values around 50 nM. Using trypan blue exclusion assay, we found that TJY-16 inhibited cell growth of C6, U87, T98G and U251 cells. Interestingly, cell death occurred in three human glioma cells whereas it was not observed in C6 rat glioma cell. Thus, we raised the dose of TJY-16 to 90 nM to determine whether cell death could be induced by high dose of TJY-16. The flow cytometric analysis revealed that G2/M arrest was still observed in contrast to failure of sub-G1 production (data not shown). However, we noted that there were numerous >4 N DNA content cell population, suggesting polyploidy or aneuploidy formation. These genomic instabilities (polyploidy and aneuploidy) have been demonstrated to be associated with mitotic catastrophe or senescence [25–27]. In the present study, we did not perform SA-beta-gal staining. In the future study, we will use SA-β-gal staining to detect abnormal spindle formation to prove mitotic catastrophe was involved in TJY-16-induced anti-tumor effects in C6 gliomas.

To examine whether glioma cell death was associated with cell cycle dysregulation, cells were stained with PI to detect DNA content for analysis of cell cycle distributions. U87 and T98G cells accumulated at G2/M phase after 12 h treatment of TJY-16 which was followed by an increase in sub-G1 phase at 24 to 48 h treatment. Recent studies have proposed that cell cycle arrest can ultimately cause apoptosis. These results suggest that TJY-16 induced G2/M arrest followed by accumulation of sub-G1 phase leading to cell death [28, 29]. In addition, U87, T98G and U251 cells exhibited chromatin condensation and elevated levels of cleaved caspase-3 in U87 cells after treatment with TJY-16 suggesting that TJY-16-induced cell death was through apoptosis.

By showing the increased levels of cleaved caspase-8 and truncated BID (tBID), we demonstrated that extrinsic apoptotic pathway was involved in TJY-16-induced apoptosis. Additionally, by JC-1 staining, we found the ΔΨm decreased in a time-dependent manner after treatment with TJY-16. The changes in ΔΨm revealed that TJY-16 caused mitochondrial dysfunction. Based on the above observations, we concluded that both extrinsic and intrinsic apoptotic pathway were elicited by TJY-16. It has been demonstrated that there is a crosstalk between extrinsic and intrinsic apoptotic pathway. BID, one of Bcl2 family members, is the key role of this “crosstalk” [30]. When extrinsic apoptotic pathway is activated, full-length BID is cleaved by caspase-8. Then, truncated BID (tBID) translocates to mitochondria and propagates apoptotic signals to mitochondria followed by intrinsic apoptotic pathway [31]. In this study, we found that both extrinsic and intrinsic apoptotic pathways were activated but the intrinsic apoptotic pathway was activated directly by TJY-16 or indirectly as a result of tBID translocation to mitochondria remains to be determined.

Our results clearly showed that TJY-16 had anti-glioma effects through G2/M arrest and apoptosis in vitro. In order to verify the effects of TJY-16 in vivo, nude mice were inoculated subcutaneously with U87 glioma cells. Treatment with TJY-16 (24 mg/kg i.p.) for continuous 10 days significantly reduced tumor volume without affecting the body weights of nude mice. Furthermore, H&E staining of the organ showed that the histological sections of brain, heart, lung, stomach, intestine and kidney were not different between TJY-16-treated and healthy mice.

TJY-16 (previously termed HAC-Y6) has been shown to display potent anti-cancer activity against hepatocellular carcinoma (HCC) and COLO205 cells in vitro through disrupting microtubule assembly, causing cell cycle arrest and apoptosis [16, 32]. Here, we for the first time demonstrated that TJY-16 was effective in inhibiting tumor growth and induced caspase-3 activation in the xenograft tumor model of U87 glioma cells. Temozolomide (TMZ) is an oral chemotherapy drug. It is an alkylating agent used as a first-line treatment for glioblastoma multiforme. Previous study has shown that TMZ at doses of 80 mg/kg or 120 mg/kg administered orally on five consecutive days significantly delay the tumor growth against brain tumor xenografts [33]. Here, we have demonstrated for the first time that TJY-16 was effective in inhibiting tumor growth and induced caspase-3 activation in the xenograft tumor model of U87 glioma cells.

## Conclusion

We have shown for the first time that TJY-16 induced G2/M cell cycle arrest and apoptosis in U87, T98G and U251 glioma cells both in vitro and in vivo. Both extrinsic and intrinsic apoptotic pathways were involved in TJY-16-induced apoptotic cell death. TJY-16 could be a promising agent for the treatment of malignant gliomas.

## Abbreviations

FACS: fluorescence-activated cell sorting
GBM: glioblastoma multiforme
ΔΨm: mitochondrial membrane potential
PI: propidium iodide
TJY-16: 6-acetyl-9-(3,4,5-trimethoxybenzyl)-9H-pyrido[2,3-b]indole
